# First genetically confirmed records of the little gulper shark *Centrophorusuyato* (Squaliformes: Centrophoridae) from Cypriot waters

**DOI:** 10.3897/BDJ.9.e71837

**Published:** 2021-09-16

**Authors:** Vasiliki Kousteni, Marios Papageorgiou, Michail Rovatsos, Ioannis Thasitis, Louis Hadjioannou

**Affiliations:** 1 Department of Ecology, Faculty of Science, Charles University, Viničná 7, 128 00, Prague, Czech Republic Department of Ecology, Faculty of Science, Charles University, Viničná 7, 128 00 Prague Czech Republic; 2 Fisheries Research Institute, Hellenic Agricultural Organization, 640 07, Nea Peramos, Kavala, Greece Fisheries Research Institute, Hellenic Agricultural Organization, 640 07 Nea Peramos, Kavala Greece; 3 Enalia Physis Environmental Research Centre, Acropoleos 2, Aglantzia 2101, Nicosia, Cyprus Enalia Physis Environmental Research Centre, Acropoleos 2, Aglantzia 2101 Nicosia Cyprus; 4 Department of Fisheries and Marine Research, Ministry of Agriculture, Natural Resources and Environment, Nicosia, Cyprus Department of Fisheries and Marine Research, Ministry of Agriculture, Natural Resources and Environment Nicosia Cyprus; 5 Cyprus Marine and Maritime Institute, CMMI House Vasileos Pavlou Square, PO Box 409 30, Larnaca, Cyprus Cyprus Marine and Maritime Institute, CMMI House Vasileos Pavlou Square, PO Box 409 30 Larnaca Cyprus

**Keywords:** Cyprus, DNA barcoding, Mediterranean Sea, Elasmobranchii, taxonomy, 16S rRNA

## Abstract

The taxonomy within the genus *Centrophorus* has been controversial almost since its origin, raising uncertainties about the identification, the phylogenetic placement and the geographical distribution of several species. The partial nucleotide sequences of two mitochondrial DNA gene regions, the cytochrome *c* oxidase subunit I and the 16S ribosomal RNA, genetically confirmed the presence of the little gulper shark in Cypriot waters. The species presence in the Mediterranean Sea is revised and discussed.

## Short communication

The family Centrophoridae Bleeker, 1859 (Chondrichthyes: Squaliformes) comprises two genera: *Centrophorus* Müller & Henle, 1837 and *Deania* Jordan & Snyder, 1902, known as gulper sharks, a group of small to medium-sized benthopelagic species that occur worldwide along the outer continental shelves and upper continental and insular slopes (Compagno 1984, Ebert and Winton 2010, Kyne and Simpfendorfer 2010). In the Mediterranean Sea, the family Centrophoridae is represented only by the genus *Centrophorus* (Compagno 1984, Ebert and Dando 2020). Both the gulper shark *Centrophorusgranulosus* (Bloch & Schneider, 1801) and the little gulper shark *Centrophorusuyato* (Rafinesque, 1810) have been included in several Mediterranean taxonomic fish checklists (e.g. Kabasakal 2002, Bilecenoğlu et al. 2002, Papaconstantinou 2014, Akel and Karachle 2017). However, recent molecular and morphometric studies (White et al. 2013, Veríssimo et al. 2014) supported the presence of a unique mitochondrial DNA (mtDNA) clade and a morphologically-distinct taxon in the Mediterranean Sea, corresponding to the small-sized species of *Centrophorus*, which erroneously was often identified as *C.granulosus*. Although we are aware that the nomenclature of the species name *C.uyato* is not definite, we follow here the recommendation of White et al. (2013) to use *C.uyato* until this taxonomical issue is resolved.

The lack of holotypes and detailed descriptions with strong diagnostic characters for *C.uyato* and *C.granulosus* has historically generated confusion over their identification (White et al. 2013, Veríssimo et al. 2014). This long-standing taxonomic issue dates back to 1906 when Garman assigned *Squalusuyato* Rafinesque, 1810 to the genus *Centrophorus* (Garman 1906), in contrast to Müller and Henle (1839) who considered it as *Acanthiasuyatus* (Müller and Henle (1839)) and Bonaparte (1841) who considered it as *Spinaxuyatus* (Bonaparte (1841)). Since then, *C.uyato* (Rafinesque, 1810) has commonly been used in the scientific literature creating historically a nomenclatural confusion, because the original description of Rafinesque's *S.uyato* is based on an undetermined species of *Squalus* and, thus, should not be used in taxonomic assignments of species of *Centrophorus* (White et al. 2013). On the other hand, although the original description of *C.granulosus* (Bloch & Schneider, 1801) is based on a large species of *Centrophorus*, which was clearly distinct from the other large congener *C.squamosus* (Bonnaterre, 1788) and formerly named as *Squalussquamosus* Bonnaterre, 1788, the re-description of *C.granulosus* by Müller and Henle (1841) was based on a small specimen from the Mediterranean Sea that represented a distinct morphotype (White et al. 2013, Veríssimo et al. 2014).

Currently, the nomenclatural validity of *C.uyato* vs *C.granulosus* remains unsettled (White et al. 2013, Veríssimo et al. 2014, Serena et al. 2020). Nevertheless, in accordance with Compagno (1984) and White et al. (2013), these species differ, based on the following main characteristics: *C.uyato* attains a smaller maximum total length (1100 mm) than *C.granulosus* (1700 mm); the denticles in *C.uyato* are flat, block-like with only a short cusp, while the denticles in *C.granulosus* are flat with teardrop-shaped crowns and a posterior cusp and are not overlapping or raised on pedicels, which gives the skin a granular texture; the first dorsal fin is short and triangular in *C.uyato*, but long with low height in *C.granulosus*; the free pectoral rear tips are moderately longer in *C.uyato* compared to equally-sized *C.granulosus* specimens.

**Figure 1.**
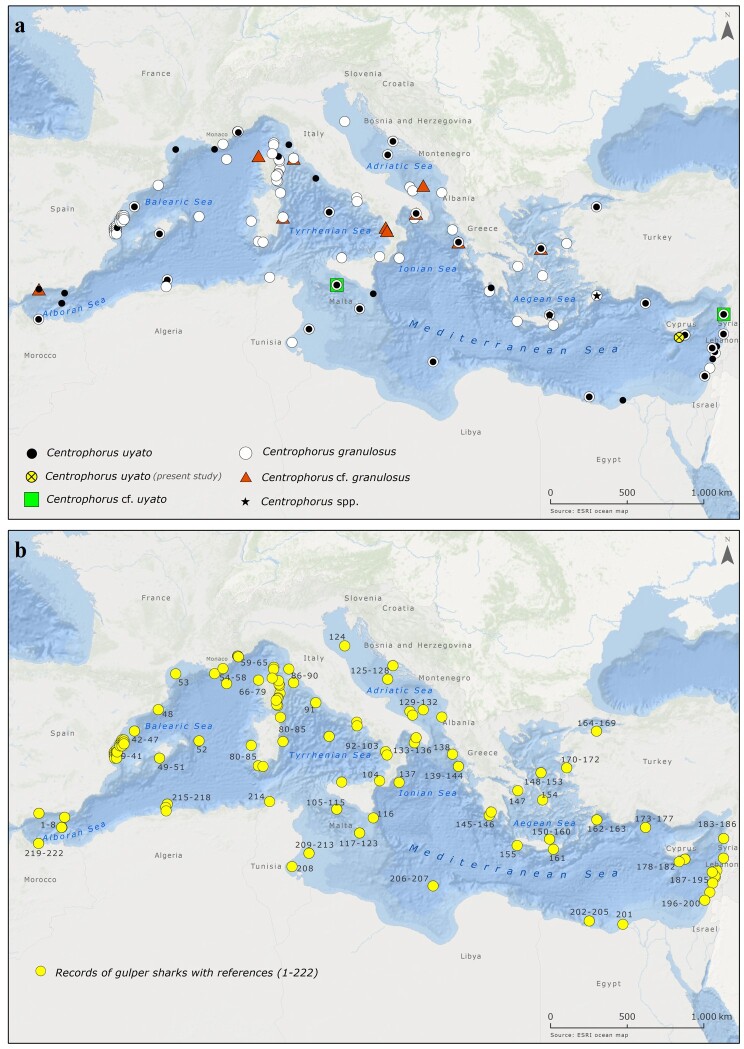
Map of the Mediterranean Sea, showing the locations of gulper sharks' records: **a**, different symbols represent the "different scientific names" used for the only species of *Centrophorus* (probably *C.uyato*) occurring in this basin, and **b**, records with references represented by the numbers 1-222 (See Suppl. material 1). The map was generated using the ArcGIS v.10.3 software.

**Figure 2.**
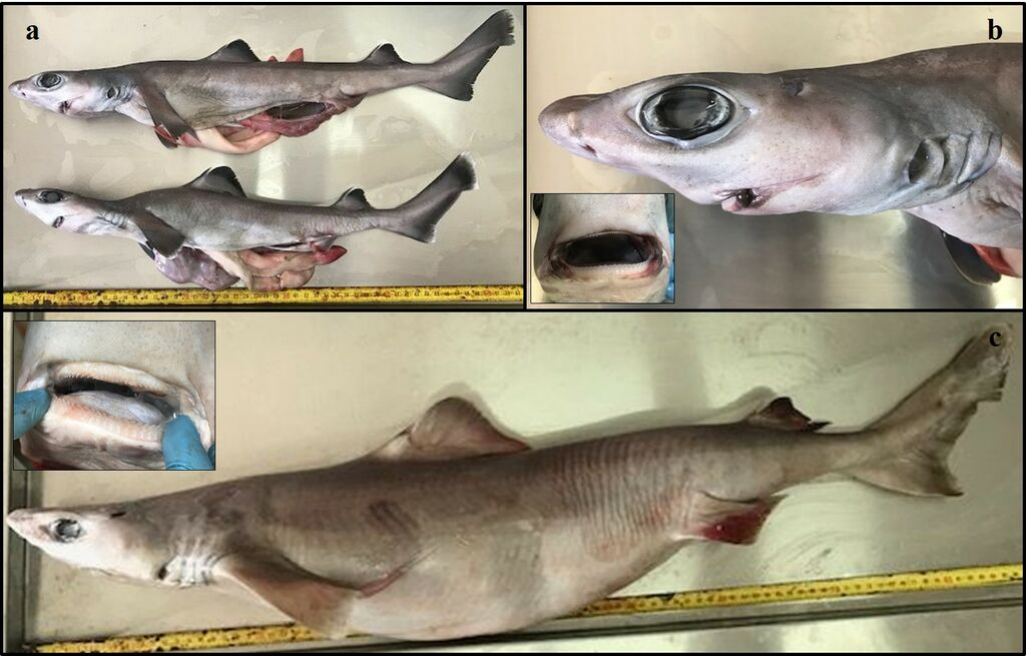
Specimens of *C.uyato* caught incidentally off southern Cyprus: **a**, lateral view of two immature females of 522 and 483 mm L_T_ **b**, profile view of the head and view of the mouth of an immature female of 522 mm L_T_, and **c**, lateral view and view of the mouth of a mature female of 890 mm L_T_.

**Figure 3.**
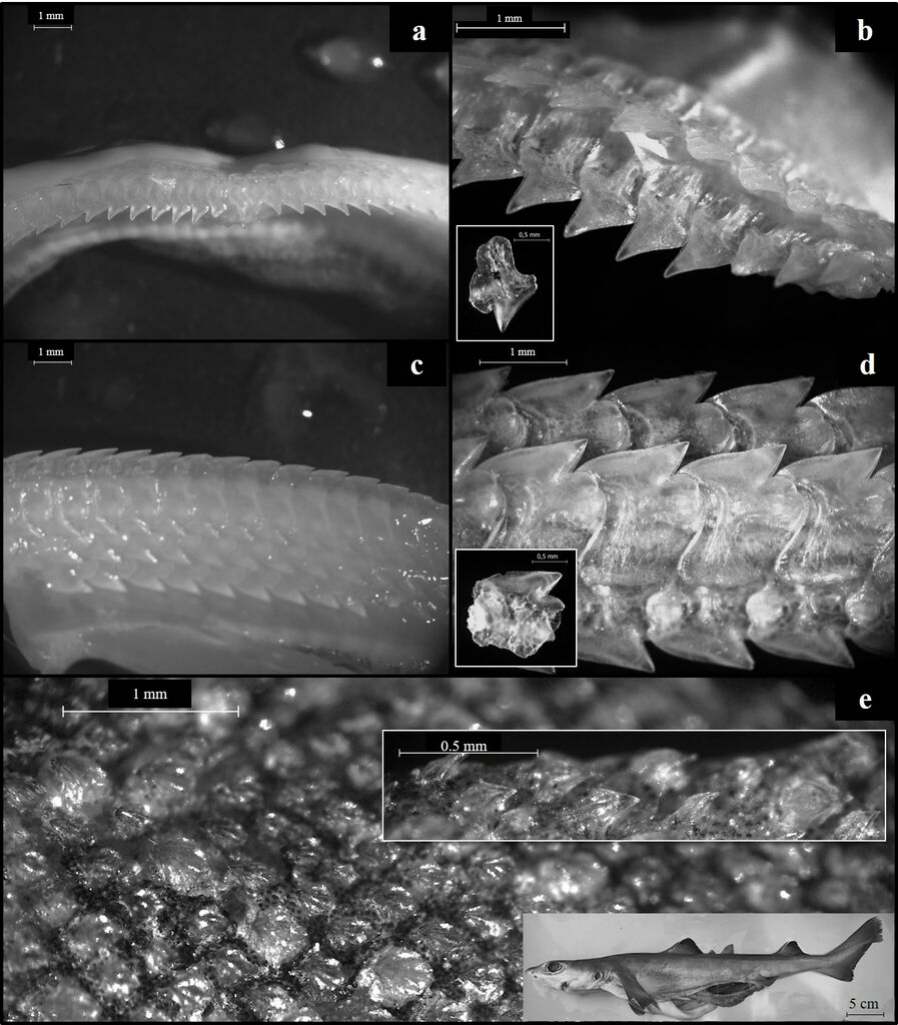
Morphometric features of an immature female *C.uyato* (503 mm L_T_) caught off southern Cyprus: **a**–**b**, upper teeth and individual tooth of the upper jaw **c**–**d**, lower teeth and individual tooth of the lower jaw, and **e**, dermal denticles. Photographs of individual teeth and of a closer view of the scales are framed in white. Individual teeth were removed and photographed following the method by Straube and Pollerspöck (2020).

On 20 July 2020, 13 little gulper sharks (Chondrichthyes: Centrophoridae) were captured during an experimental bottom trawl survey off the southern coast of Cyprus (geographical position: 34°21'25"N, 33°07'11"E) at 605 m depth (Fig. 1, Suppl. material 1). All specimens were landed dead on board and were kept for further examination. Sampling was conducted by the Cypriot National Data Collection Programme, under the European Community Data Collection Framework (Regulations EC2017/1004, 665/2008 and Decisions 2019/909, 2019/910, 2019/910) following the Mediterranean International Bottom Trawl Survey (MEDITS) Handbook (Anonymous 2017). Total length (L_T_; mm) was measured from the tip of the snout to the tip of the upper caudal fin. Total mass (M_T_; g) was recorded as the total weight of each specimen. Following Compagno (1984), a total of 83 morphometric measurements (including L_T_) were recorded in two immature female individuals. In each individual, the sex was determined and the maturity stage was assessed macroscopically following the maturity scales specialised in Squaliformes (Stehmann 1987, McLaughlin and Morrissey 2005, Kousteni and Megalofonou 2011). Following Compagno (1984) and White et al. (2013), the macroscopic characteristics of all specimens resembled those of *C.uyato* (Figs 2, 3). Molecular methods were used as a complementary tool for species identification as commonly applied in elasmobranch research (Ward et al. 2005, Kousteni et al. 2016, Kousteni et al. 2021). For this purpose, individual fin clips were obtained from all 13 individuals, preserved in 95% ethanol and stored at -20°C.

Genomic DNA was extracted from approximately 25 mg of each fin sample using the standard protocol of the DNeasy Blood and Tissue Kit (Qiagen, Chatsworth, CA, USA). The DNA concentration of each sample was estimated using a NanoDrop One Spectrophotometer (Thermo Scientific, Wilmington, DE, United States). DNA fragmentation was checked using a 1% agarose gel electrophoresis. Following, two mtDNA gene regions, the 652 bp fragment of the cytochrome oxidase *c* subunit I (COI) and 580 bp fragment of the 16S ribosomal RNA (16S rRNA) were amplified in each of the 13 specimens using polymerase chain reaction (PCR) with the following sets of primers: FishF2 5'-TCGACTAATCATAAAGATATCGGCAC-3', FishR2 5'-ACTTCAGGGTGACCGAAGAATCAGAA-3' for COI (Ward et al. 2005) and 16SarL 5'-CGCCTGTTTATCAAAAACAT3', 16SbrH 5'-CCGGTCTGAACTCAGATCACGT-3 for 16S rRNA (Palumbi et al. 1991). The fragments were amplified separately for each specimen. 25 μl PCR mixtures for both primer sets contained 0.5 μl DNA template (50–100 ng/μl), 18.5 μl ultra-pure water, 2.5 μl 10x PCR buffer (BioTaq, Bioline), 1.25 μl MgCl_2_ (50 mM), 1 μl dNTPs (10 mM), 0.5 μl of each primer (10 mM) and 0.25 U Taq DNA polymerase (BioTaq, Bioline). The PCR amplification conditions for both gene fragments were as follows: an initial denaturation of 2 min at 95°C, followed by 35 cycles of 30 s for denaturation at 94°C, 45 s for the annealing of primers at 54°C, 45 s for the extension of fragments at 72°C and a final extension step for 10 min at 72°C. The PCR products (1 μl) were visualised by electrophoresis on a 1% agarose gel. Successful amplicons were sequenced bi‐directionally by Macrogen Europe (Amsterdam, The Netherlands).

The obtained mtDNA sequences were imported into Geneious Prime software (Kearse et al. 2012) and checked for quality and accuracy in nucleotide base assignment. The comparison of the sequences revealed a single haplotype in both mtDNA gene regions for all 13 examined individuals. For cross-species comparisons, the taxonomically revised dataset of Veríssimo et al. (2014) was used and both the COI and the 16S rRNA sequences of specimens of *Centrophorus* were obtained from GenBank (Suppl. material 2). In total, 32 haplotypes of the COI gene region and 15 haplotypes of the 16S rRNA gene region of seven species of *Centrophorus* were aligned using the CLUSTAL W algorithm (Higgins 1994) and the birdbeak dogfish *Deaniacalcea* (Lowe, 1839) as an outgroup. The mean pairwise genetic distances between the species and the intraspecific distances within species haplo-groups (Suppl. material 3) were calculated using MEGA v.10 software (Kumar et al. 2018). MEGA was also used to construct a Neighbour-Joining (NJ) tree with 1000 bootstrap replicates as statistical support.

**Figure 4.**
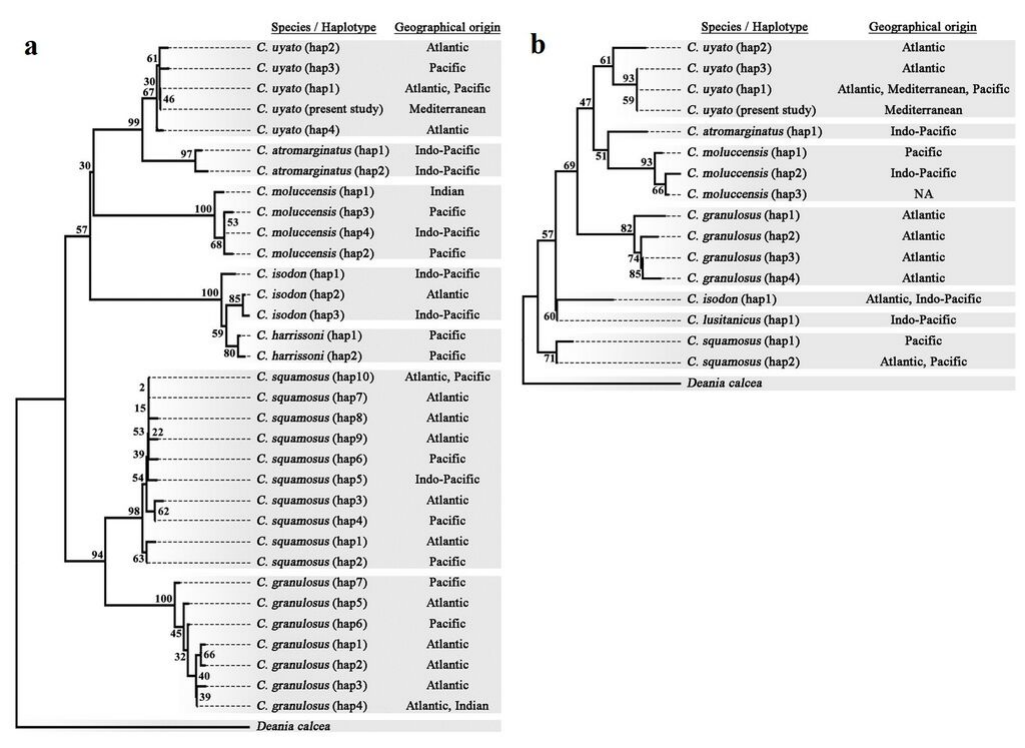
Neighbour-Joining tree of the mean genetic *p*-distances amongst the mtDNA haplotypes for **a**, the COI and **b**, the 16S rRNA gene regions of species of *Centrophorus*, based on the revised taxonomic assessment of Veríssimo et al. (2014) (Suppl. material 2). Support values (%) for each clade, based on 1000 bootstrap replicates, are indicated on the top of each branch.

In the present study, no genetic polymorphism was found amongst all 13 individuals and a single mtDNA haplotype was generated for either the COI (GenBank Assession Numbers: MZ456040-MZ456052) or the 16S rRNA gene region (GenBank Assession Numbers: MZ452674-MZ452686). Each mtDNA haplotype was grouped with the *C.uyato* cluster (Fig. 4), therefore genetically confirming the occurrence of the little gulper shark in Cypriot waters. In the region, gulper sharks have been reported as *C.granulosus* (Hadjichristophorou 2006, EU DCF CYP MEDITS 2009), probably corresponding to the small species of *Centrophorus* that occurs in the Mediterranean Sea (Veríssimo et al. 2014, Serena et al. 2020). Our data support the recommendation of White et al. (2013) to classify the small species of the genus *Centrophorus*, which erroneously was often referred to as *C.granulosus*, as *C.uyato* and enhance the genetic results of Veríssimo et al. (2014) supporting a unique mtDNA clade for the genus *Centrophorus* in the Mediterranean Sea.

The occurrence of *C.uyato* in the Mediterranean Sea and the adjacent Atlantic Ocean can be considered as verified (White et al. 2013). Recent molecular and morphological data (Wienerroither et al. 2015) have also shown that *C.uyato* is conspecific to *Centrophoruszeehaani*, which is endemic to southern Australia (White et al. 2008), thus supporting the occurrence of *C.uyato* in the Pacific Ocean. On the other hand, *C.granulosus* has a wider circumglobal distribution in tropical and temperate seas (Fricke et al. 2021). Due to the misidentification of *C.uyato* with *C.granulosus*, the overall distribution of the species remains uncertain (Wienerroither et al. 2015, Ebert and Dando 2020). According to the available scientific literature, the distribution of *C.uyato* in the Mediterranean Sea is shown in Fig. 1 along with the species records under "different" scientific names, highlighting the nomenclatural confusion around this species. For the same reason, the available information for the species biology is limited (McLaughlin and Morrissey 2005, Lteif et al. 2017). In Cypriot waters, females ranged between 375 – 965 mm L_T_ (Mean ± S.D = 653 ± 213 mm L_T_) and 575 – 5800 g M_T_ (Mean ± S.D = 2329 ± 2377 g M_T_) and males ranged from 730 to 860 mm L_T_ (Mean ± S.D = 803 ± 54 mm L_T_) and from 2330 to 3730 g M_T_ (Mean ± S.D = 3290 ± 647 g M_T_). The morphometric measurements of two immature female individuals are presented in Suppl. material 4. Although females reached larger body size than males, significant between-sex differences were not found in the median values of L_T_ and M_T_ (Mann-Whitney test: W = 24 and P > 0.05 in both cases), probably because of the small sample size. The total mass-total length relationship for sexes combined is described by the equation: M_T_ = 4E-06 L_T_^3.0585^ (R^2^ = 0.93) indicating positive allometric growth. Six females were immature ranging from 375 to 599 mm L_T_, while 3 females between 890 – 965 mm L_T_ were mature with either large yellow oocytes, embryos in their oviducts or enlarged and empty oviducts. All males (n = 4) were mature, either sexually active or at resting phase.

Over the last 50 years, the alpha taxonomy within the genus *Centrophorus* has been extensively revised, resulting in provisional conclusions (Bigelow and Schroeder 1957, Naylor et al. 2012, White et al. 2008, White et al. 2017, White et al. 2013) and implying that the distribution range of several species remains uncertain (Bañón et al. 2008, Kyne and Simpfendorfer 2010). Herein, we present all the known-to-date records of the gulper sharks in the Mediterranean Sea, probably referred to as *C.uyato*, which is the only verified gulper shark in this region (Ebert and Dando 2020, Serena et al. 2020), based on the available scientific literature and the web service of the Global Biodiversity Information Facility (GBIF, https://www.gbif.org/) (Fig. 1). Nevertheless, the overall distribution of *C.uyato* needs revision as soon as a definite taxonomic assessment is achieved.

In conclusion, we would like to stress the need to establish an international network of experts with the scope to implement a holistic taxonomic assessment for the gulper sharks by applying both molecular and morphometric tools in a sufficient number of specimens per species representing all ontogenetic stages and different locations. This effort, apart from achieving a definite taxonomic assessment, will redirect fisheries statistics towards the proper management of *C.uyato* and *C.granulosus*. Furthermore, considering that, according to IUCN, both species are listed as Endangered (EN) globally, *C.uyato* is unassessed in the Mediterranean and *C.granulosus* is listed as Critically Endangered (CR) for the region, in conjunction with the fact that all Mediterranean records of *C.granulosus* may be incorrect, the re-asessment of the species extinction risk should be prioritised as new taxonomical-distribution data are becoming available. The correct identification throughout the species distribution range will minimise the potential threat to both species and will direct future efforts within the IUCN for the successful conservation of their population stocks.

## Supplementary Material

4E8FA947-3DDE-50FB-97CA-12DE5EE3F9B3 10.3897/BDJ.9.e71837.suppl1 Supplementary material 1 Number (N) of studies reporting gulper sharks in the Mediterranean Sea Data type occurrences Brief description Number (N) of studies reporting gulper sharks in the Mediterranean Sea. The scientific name/s reported in each study are provided. File: oo_568503.xlsx https://binary.pensoft.net/file/568503 Vasiliki Kousteni, Marios Papageorgiou, Michail Rovatsos, Ioannis Thasitis, Louis Hadjioannou

407A3F52-0D74-5835-8952-775F72436AE3 10.3897/BDJ.9.e71837.suppl2 Supplementary material 2 List of haplotype groups of species of *Centrophorus* included in the Neighbour-Joining analysis Data type haplotype groups Brief description List of haplotype groups (hap) of species of *Centrophorus* for each mtDNA gene region, cytochrome oxidase *c* subunit I (COI) and 16S ribosomal RNA (16S rRNA), included in the Neighbour-Joining analysis. The haplotype groups are based on the revised species designation dataset of Veríssimo et al. (2014). The sequences generated in the present study are indicated in bold. File: oo_586171.xlsx https://binary.pensoft.net/file/586171 Vasiliki Kousteni, Marios Papageorgiou, Michail Rovatsos, Ioannis Thasitis, Louis Hadjioannou

D1189F8D-B7ED-58DB-9EB4-3FB52B222755 10.3897/BDJ.9.e71837.suppl3 Supplementary material 3 Mean pairwise genetic *p*-distances (below the diagonal) and intraspecific distances (in bold) between mtDNA COI and 16S rRNA haplotypes of species of *Centrophorus* Data type genetic p-distances Brief description Mean pairwise genetic *p*-distances (%*p*, below the diagonal) between seven species of *Centrophorus* for the mtDNA COI and 16S rRNA gene regions. The intraspecific genetic distances are indicated in bold. File: oo_585993.xlsx https://binary.pensoft.net/file/585993 Vasiliki Kousteni, Marios Papageorgiou, Michail Rovatsos, Ioannis Thasitis, Louis Hadjioannou

A9B34EC2-7E0B-5811-9FBD-73ECBEB89594 10.3897/BDJ.9.e71837.suppl4 Supplementary material 4 Measurements of 83 morphometric characteristics taken in a sub-sample of *C.uyato* off southern Cyprus following Compagno (1984) Data type morphometric measurements Brief description Morphometric measurements of two immature female little gulper sharks caught incidentally off southern Cyprus. Values are expressed in mm and as percentages of the total length (%L_T_). File: oo_568380.pdf https://binary.pensoft.net/file/568380 Vasiliki Kousteni, Marios Papageorgiou, Michail Rovatsos, Ioannis Thasitis, Louis Hadjioannou
