# Supplementary material for: First genetically confirmed records of the little gulper shark Centrophorusuyato (Squaliformes: Centrophoridae) from Cypriot waters
Source: Biodivers Data J. 2021 Sep 16;9:e71837. doi: 10.3897/BDJ.9.e71837 (PMC8460593; doi:10.3897/BDJ.9.e71837)
Supplement: Supplementary material 4 — Measurements of 83 morphometric characteristics taken in a sub-sample of C.uyato off southern Cyprus following Compagno (1984) [file bdj-09-e71837-s004.pdf]

Suppl. material 4. Morphometric measurements of two female little gulper sharks caught incidentally off southern Cyprus. Values are expressed in mm and as percentages of the total length (%L_T).

**Morphometric characteristics**	**Specimen 1**		**Specimen 2**	
	**in mm**	**as %L_T**	**in mm**	**as %L_T**
Total length (TL)	522.00		483.00	
Fork length (FL)	458.00	87.74	415.00	85.92
Pre-orbital length (POB)	38.86	7.44	33.31	6.90
Pre-spiracular length (PSP)	76.54	14.66	68.12	14.10
Pre-branchial length (PGI)	107.02	20.50	97.58	20.20
Head length (HDL)	129.56	24.82	115.05	23.82
Pre-first dorsal length (PD1)	170.18	32.60	155.65	32.23
Pre-second dorsal length (PD2)	331.50	63.51	307.62	63.69
Pre-caudal length (PRC)	415.00	79.50	382.12	79.11
Interdorsal space (IDS)	105.81	20.27	96.8	20.04
Dorsal caudal space (DCS)	41.21	7.89	33.31	6.90
Pre-pectoral length (PP1)	125.52	24.05	118.18	24.47
Pre-pelvic length (PP2)	303.68	58.18	293.11	60.69
Pectoral-pelvic space (PPS)	148.70	28.49	144.05	29.82
Pelvic-caudal space (PCA)	75.61	14.48	61.19	12.67
Snout-vent length (SVL)	319.09	61.13	308.62	63.90
Vent-caudal length (VCL)	202.91	38.87	174.38	36.10
First dorsal length (D1L)	90.76	17.39	80.66	16.70
First dorsal anterior margin (D1A)	63.44	12.15	49.66	10.28
First dorsal base (D1B)	60.26	11.54	55.52	11.49
First dorsal height (D1H)	33.23	6.37	30.46	6.31
First dorsal inner margin (D1I)	30.50	5.84	25.14	5.20
First dorsal posterior margin (D1P)	45.33	8.68	40.12	8.31
Second dorsal length (D2L)	64.52	12.36	61.31	12.69
Second dorsal anterior margin (D2A)	48.16	9.23	44.11	9.13
Second dorsal base (D2B)	42.29	8.10	41.19	8.53
Second dorsal height (D2H)	20.14	3.86	20.66	4.28
Second dorsal inner margin (D2I)	22.26	4.26	20.12	4.17
Second dorsal posterior margin (D2P)	33.18	6.36	26.27	5.44
Pectoral length (P1L)	32.06	6.14	30.72	6.36
Pectoral anterior margin (P1A)	60.71	11.63	54.89	11.36
Pectoral base (P1B)	29.80	5.71	28.28	5.86
Pectoral height (P1H)	58.57	11.22	53.28	11.03
Pectoral inner margin (P1I)	61.11	11.71	57.72	11.95
Pectoral posterior margin (P1P)	54.60	10.46	50.86	10.53
Pelvic length (P2L)	54.40	10.42	47.83	9.90
Pelvic anterior margin (P2A)	34.01	6.52	32.39	6.71
Pelvic base (P2B)	25.30	4.85	21.04	4.36
Pelvic height (P2H)	22.51	4.31	21.63	4.48
Pelvic inner margin (P2I)	30.46	5.84	28.29	5.86
Pelvic posterior margin (P2P)	28.59	5.48	26.64	5.52
Dorsal caudal margin (CDM)	106.69	20.44	99.61	20.62
Pre-ventral caudal margin (CPV)	67.28	12.89	55.66	11.52
Upper postventral caudal margin (CPU)	43.89	8.41	36.3	7.52
Lower postventral caudal margin (CPL)	16.11	3.09	14.9	3.08
Caudal fork width (CFW)	40.75	7.81	33.18	6.87
Caudal fork length (CFL)	64.09	12.28	54.7	11.33
Subterminal caudal margin (CST)	16.17	3.10	15.58	3.23
Subterminal caudal width (CSW)	26.57	5.09	25.16	5.21
Terminal caudal margin (CTR)	28.73	5.50	27.62	5.72

Suppl. material 4 - Continued				
**Morphometric characteristics**	**Specimen 1**		**Specimen 2**	
	**in mm**	**as %L_T**	**in mm**	**as %L_T**
Terminal caudal lobe (CTL)	33.82	6.48	32.74	6.78
Pre-narial length (PRN)	24.56	4.70	20.73	4.29
Pre-oral length (POR)	53.92	10.33	44.87	9.29
Eye length (EYL)	32.89	6.30	31.57	6.54
Eye height (EYH)	9.03	1.73	8.56	1.77
Intergill length (ING)	22.54	4.32	17.47	3.62
First gill slit height (GS1)	9.50	1.82	8.67	1.80
Second gill slit height (GS2)	10.05	1.93	9.51	1.97
Third gill slit height (GS3)	10.78	2.07	9.98	2.07
Fourth gill slit height (GS4)	11.54	2.21	11.42	2.36
Fifth slit height (GS5)	12.42	2.38	12.39	2.57
Mouth length (MOL)	17.90	3.43	13.15	2.72
Mouth width (MOW)	42.29	8.10	33.36	6.91
Nostril width (NOW)	11.51	2.20	8.46	1.75
Internarial space (INW)	20.42	3.91	16.58	3.43
Anterior nasal flap length (ANF)	4.25	0.81	3.08	0.64
Interorbital space (INO)	41.23	7.90	39.96	8.27
Spiracle length (SPL)	6.64	1.27	5.15	1.07
Eye spiracle space (ESL)	10.42	2.00	7.48	1.55
Head width (HDW)	58.24	11.16	48.14	9.97
Trunk width (TRW)	63.95	12.25	58.59	12.13
Abdomen width (ABW)	50.16	9.61	42.51	8.80
Tail width (TAW)	25.82	4.95	21.83	4.52
Caudal peduncle width (CPW)	11.32	2.17	10.10	2.09
Head height (HDH)	37.91	7.26	31.03	6.42
Trunk height (TRH)	54.44	10.43	42.16	8.73
Abdomen height (ABH)	49.86	9.55	45.11	9.34
Tail height (TAH)	33.18	6.36	31.92	6.61
Caudal peduncle length (CPH)	18.04	3.46	15.93	3.30
Pelvic midpoint-first dorsal insertion (PDI)	72.84	13.95	67.76	14.03
Pelvic midpoint-second dorsal origin (PDO)	28.36	5.43	26.85	5.56
First dorsal midpoint-pectoral insertion (DPI)	59.98	11.49	56.21	11.64
First dorsal midpoint-pelvic origin (DPO)	86.06	16.49	79.55	16.47
